# Supplementary material for: Ankyrin domain encoding genes from an ancient horizontal transfer are functionally integrated into Nasonia developmental gene regulatory networks
Source: Genome Biol. 2018 Sep 28;19:148. doi: 10.1186/s13059-018-1526-x (PMC6161386; doi:10.1186/s13059-018-1526-x)
Supplement: Supplementary file 1 — Table S1. Reference sequences and names corresponding to each novel ankyrin-repeat containing Nasonia transcripts. Table S2. CLANK ortholog candidate sequences in Melittobia. Table S3. Nasonia off-target hits from Melittobia CLANK ortholog BLAST. Table S4. Melittobia primer sequences for in situ hybridization probes. Figure S1. Phylogenetic analysis of Nasonia DV-regulated CLANK protein family. Figure S2. CLANKs lacking differential expression patterns. Figure S3. Distribution of pRNAi survival rate for each CLANK of interest. Figure S4. Relative embryonic expression of CLANK transcripts over time following pRNAi. Figure S5. Effects of reducing CLANKs on Nv-zen expression. Figure S6. Effects of reducing CLANKs on Nv-twi expression. Figure S7. Melittobia CLANK candidates lacking differential expression patterns. (PDF 11494 kb) [file 13059_2018_1526_MOESM1_ESM.pdf]

		NCBI Reference Sequence		
Working Code	Transcript	Gene Symbol	Transcripts	Proteins
A	Nasvi2EG001954	LOC103316295	XM_016985565.1	XP_016841054.1
-	-	-	XM_008209433.2	XP_008207655.1
B	Nasvi2EG004695	LOC100678068	XM_003423962.3	XP_003424010.1
C	Nasvi2EG004073	LOC107980632	XM_016981576.1	XP_016837065.1
D	Nasvi2EG001932	LOC107981098	XM_016985581.1	XP_016841070.1
-	-	-	XM_016985580.1	XP_016841069.1
E	Nasvi2EG022697	LOC103315552	XM_008204996.2	XP_008203218.1
F	Nasvi2EG001522	LOC103316271	XM_008209395.2	XP_008207617.1
G	Nasvi2EG006715	LOC107981233	XM_016986485.1	XP_016841974.1
H	Nasvi2EG001594	LOC103316276	XM_016985613.1	XP_016841102.1
-	-	-	XM_008209403.2	XP_008207625.1
I	Nasvi2EG000245	LOC103317795	XM_008217050.2	XP_008215272.1
J	Nasvi2EG013987	LOC103316722	XM_008211962.2	XP_008210184.1
K	Nasvi2EG005791	LOC107982166	XM_016989714.1	XP_016845203.1
L	Nasvi2EG020516	LOC107981188	XM_016986174.1	XP_016841663.1
M	Nasvi2EG004500	LOC103317447	XM_008215555.1	XP_008213777.1
N	Nasvi2EG002650	LOC103316675	XM_008211709.1	XP_008209931.1
O	Nasvi2EG003104	LOC100678575	XM_003427451.3	XP_003427499.1

Additional File 1: Table S1. Reference sequences and names corresponding to each novel ankyrin-repeat containing *Nasonia* transcripts. "Working Code", "Transcript" number from annotation 2.0 of the *N. vitripennis* genome, and corresponding "Gene Symbol", "NCBI Reference Sequence" (mRNA), and "NCBI Reference Sequence" (protein) for each transcript of interest. Alternatively, spliced transcripts and proteins are listed as a second row for a given "Transcript."

Working Code	*Melittobia*
A	TRINITY DN55303 c0 g1 i1
B	TRINITY DN18414 c1 g1 i7
C	TRINITY DN18387 c4 g1 i2
D	TRINITY DN18259 c6 g3 i3
E	TRINITY DN16811 c0 g1 i6
E2	TRINITY DN16811 c0 g1 i4
F	TRINITY DN16425 c0 g1 i10
F2	TRINITY DN16425 c0 g1 i2
G	TRINITY DN16149 c0 g1 i4
H	TRINITY DN15814 c0 g1 i8
I	TRINITY DN14334 c0 g1 i2
I2	TRINITY DN14334 c0 g1 i1
J	TRINITY DN12682 c0 g1 i1
K	TRINITY DN11450 c0 g2 i1
L	TRINITY DN10211 c0 g2 i1
M	TRINITY DN8538 c0 g1 i1
N	TRINITY DN5297 c0 g1 i1

Additional File 1: Table S2: CLANK ortholog candidate sequences in *Melittobia*. Column A: Working code used throughout paper to identify sequences. Column B: *Melittobia* CLANK sequence accession numbers from de novo embryonic transcriptome (in prep).

Working Code	*Nasonia*	NCBI Ref. Seq.
Nv-1	Nasvi2EG004020t1	XP_008212763.2
Nv-2	Nasvi2EG004874t1	XP_008212763.2
Nv-3	Nasvi2EG005974t2	XP_003428174.2
Nv-4	Nasvi2EG006469t1	XP_016841663.1
Nv-5	Nasvi2EG010051t5	XP_016837448.1
Nv-6	Nasvi2EG011017t1	XP_003426146.1
Nv-7	Nasvi2EG011332t1	XP_008216264.1
Nv-8	Nasvi2EG011990t2	XP_016838884.1
Nv-9	Nasvi2EG014458t1	XP_001603925.1
Nv-10	Nasvi2EG015085t2	XP_016837069.1
Nv-11	Nasvi2EG021417t1	XP_008216036.1

Additional File 1: Table S3: *Nasonia* off-target hits from *Melittobia* CLANK ortholog BLAST. Column A: Working code used throughout paper to identify sequences. Column B: *Nasonia* sequence accession numbers from annotation 2.0 of the *N. vitripennis* genome. Column C: *Nasonia* NCBI Reference Sequences.

Primer Name	Primer Sequence
Mdig_Ank_AF.2	ggccgcggTGCAATATCGAGCTGCTGAG
Mdig_Ank_AR.2	cccggggcTGAGCACGACTCTTTTGGTG
Mdig_Ank_BF.2	ggccgcggAAGGCGGTCAATTATTGTGG
Mdig_Ank_BR.2	cccggggcTACCGCAACTGTCGAATGAA
Mdig_Ank_CF.2	ggccgcggATTGAAGGCCAACGATGAAG
Mdig_Ank_CR.2	cccggggcAGTCGTGAACGCTAGCACCT
Mdig_Ank_DF	ggccgcggATGGCCTGACTCCGTTACAC
Mdig_Ank_DR	cccggggcCACTTCTTCTTGCGCTTCCT
Mdig_Ank_EF	ggccgcggACTACAATGCCGCCCAAAT
Mdig_Ank_ER	cccggggcAGCAGGGTGTCGATCAGTTC
Mdig_Ank_E2F	ggccgcggCACGACAAAAGCTTCAACGA
Mdig_Ank_E2R	cccggggcGTCGAGCACCTCCTCTTCAC
Mdig_Ank_FF	ggccgcggTGACGGCCAGAATATCCTTC
Mdig_Ank_FR	cccggggcCGTCAGCTCCAGCTTTTACC
Mdig_Ank_F2F	ggccgcggTGTGGATTTACGCCGTTACA
Mdig_Ank_F2R	cccggggcCAAGCGTACTTAGCCGGTTC
Mdig_Ank_GF.2	ggccgcggCGATGGTAATGGGAATACCG
Mdig_Ank_GR.2	cccggggcTTCGTTCATGGAAGGATGTG
Mdig_Ank_HF	ggccgcggAGAATACCGCTGGTCTGCAT
Mdig_Ank_HR	cccggggcTCGCTTCTCGGATACCTGTT
Mdig_Ank_IF.2	ggccgcggATGGACGTCAAAGGGTTGAG
Mdig_Ank_IR.2	cccggggcAGCGCCAAGGTTTAGCAATA
Mdig_Ank_I2F.2	ggccgcggATGGACGTCAAAGGGTTGAG
Mdig_Ank_I2R.2	cccggggcGGAGTTCTGCCGTGTTTGTT
Mdig_Ank_JF	ggccgcggTGGGGCAAGAGTTAATGACA
Mdig_Ank_JR	cccggggcCTCGATCCTGCTTCATCCTC
Mdig_Ank_KF.2	ggccgcggCGGACAGTCATGGTGTAACG
Mdig_Ank_KR.2	cccggggcACTATCCCAATCGCCAAACA
Mdig_Ank_LF.2	ggccgcggGCTGTTGAATGGGGAAGAGA
Mdig_Ank_LR.2	cccggggc TCTTTGCTGTCAATGCTTGC
Mdig_Ank_MF.2	ggccgcggTCGTTGCTGTGCTATTGGAG
Mdig_Ank_MR.2	cccggggcGTCAATCCAGATTCGGTGGT
Mdig_Ank_NF	ggccgcggAGCTGGATGGACACCAGTTC
Mdig_Ank_NR	cccggggcCGACAAGTGCAATGGAGTGT

Additional File 1: Table S4: *Melittobia* primer sequences for *in situ* hybridization probes. Column A: Primer name. Column B: Primer sequence. Primers were designed using Primer3 v.0.4.0 (**http://primer3.ut.ee**) and synthesized by Integrated DNA Technologies (IDT, www.idtdna.com/Site/Order/oligoentry).

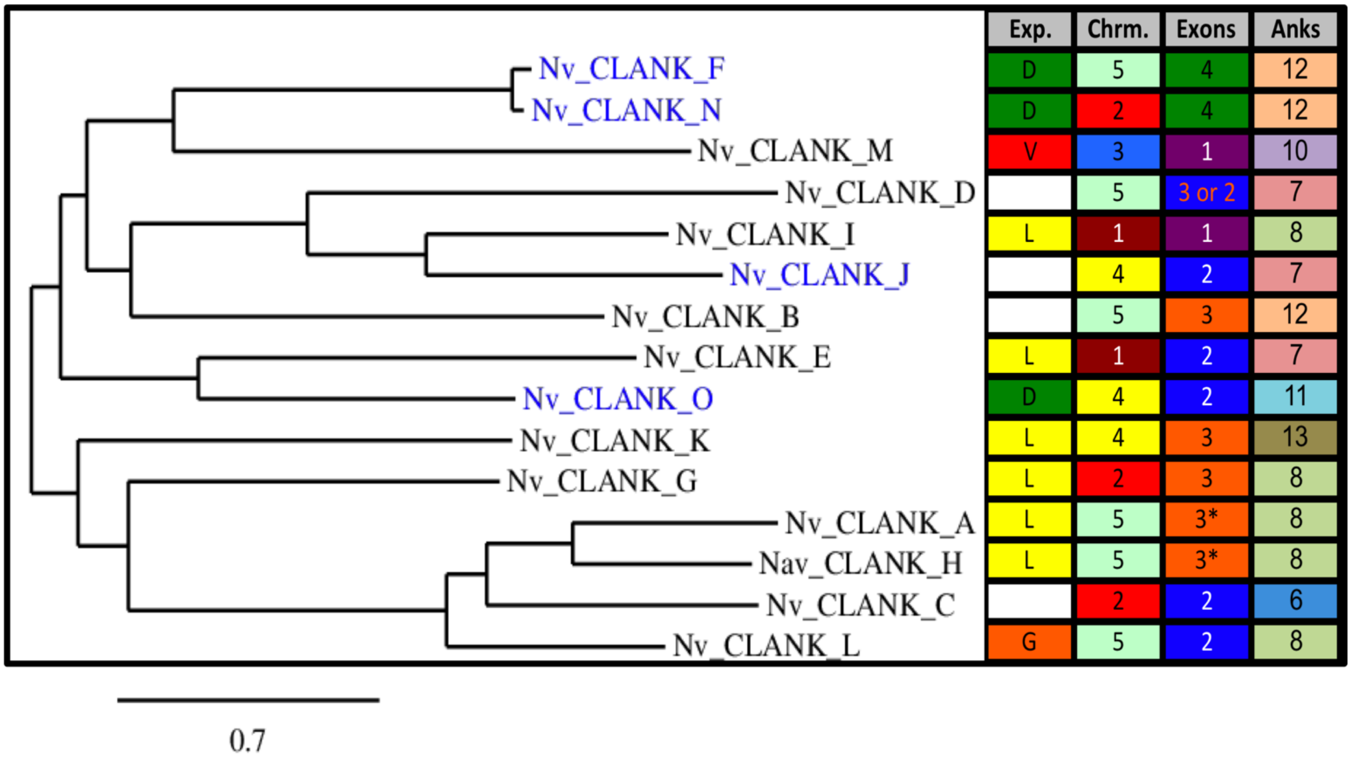

Additional File 1: Figure S1: Phylogenetic analysis of *Nasonia* DV regulated CLANKs protein family. (Left) Phylogenetic tree of CLANK proteins of interest generated using "One Click" Phylogeny Analysis (http://phylogeny.lirmm.fr/) [25]. Branch length is proportional to the number of substitutions per site. Blue text represents proteins containing a PRANC domain. (Right) Corresponding mRNA expression domain (D = dorsally, L = laterally, V = ventrally, G = no diff. expression until gastrulation, blank = ubiquitous or lack of expression), chromosomal location, number of exons (* = two differentially spliced transcripts occur, both contain same number of exons), and number of ankyrin repeats for each CLANK protein of interest. Colors added to emphasize similar value in each column.

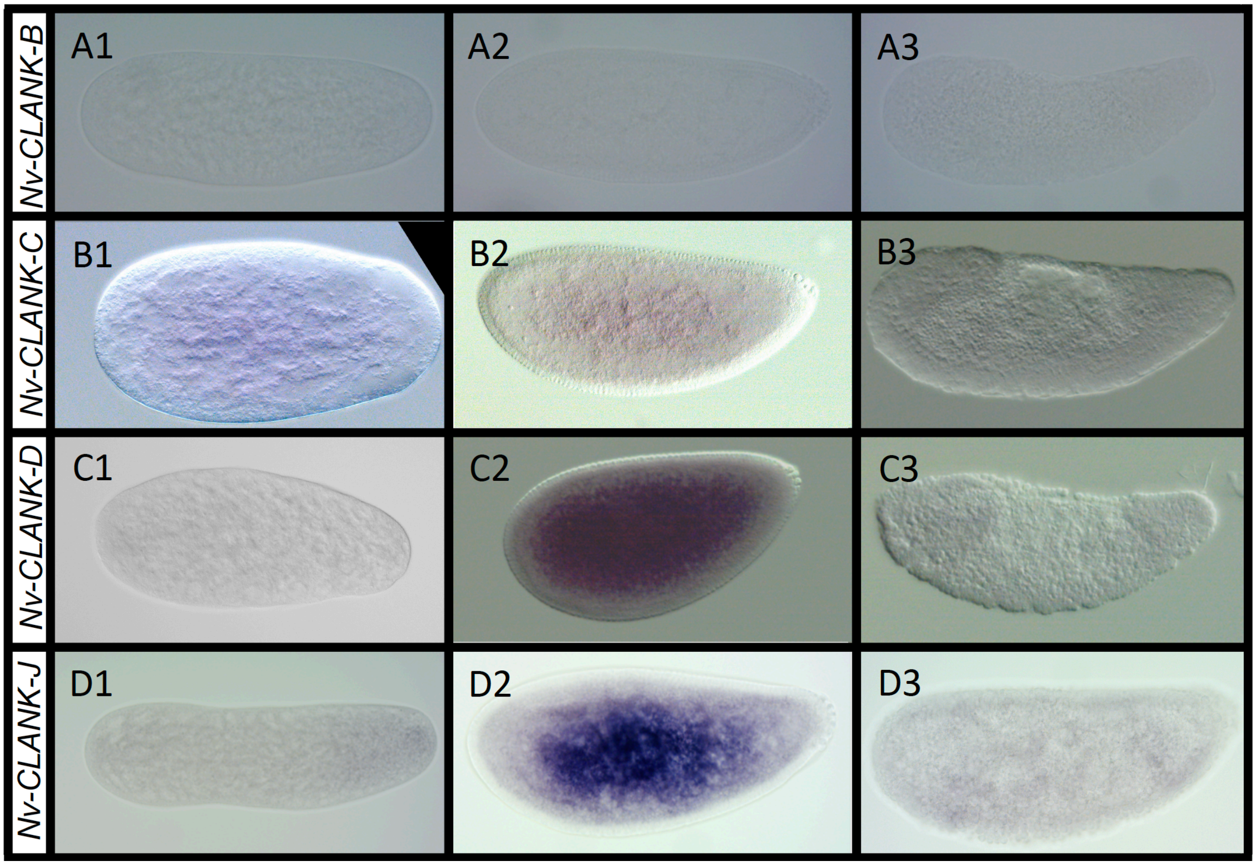

Additional File 1: Fig. S2. CLANKs lacking differential expression patterns. A1-D3 Expression of *Nv-CLANK-B*, *-C*, *-D*, *-J* from pre-blastoderm through gastrulation. All embryos are oriented with anterior to the left, posterior to the right, dorsal up, and ventral down.

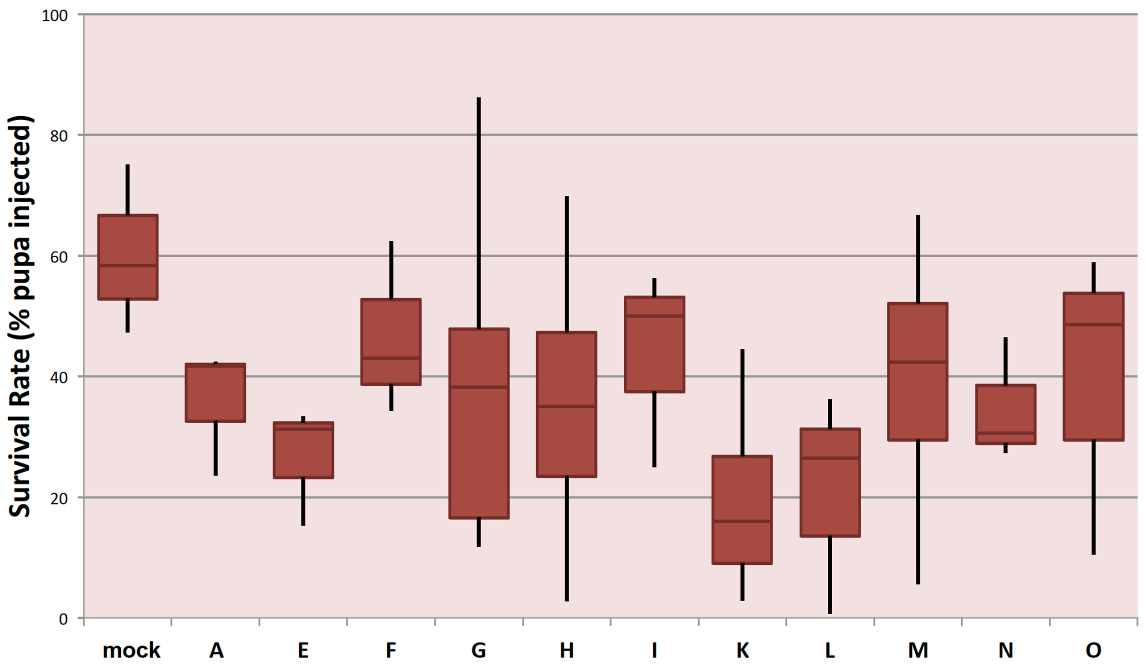

Additional File 1: Fig. S3. Distribution of pRNAi survival rate for each CLANK of interest. Range of pupal survival and eclosure (as a percentage) observed in pRNAi knockdown females for each CLANK and mock injection. Error bars represent minimal and maximum values. Horizontal line represents median value. Red box ranges from lower to upper quartile values.

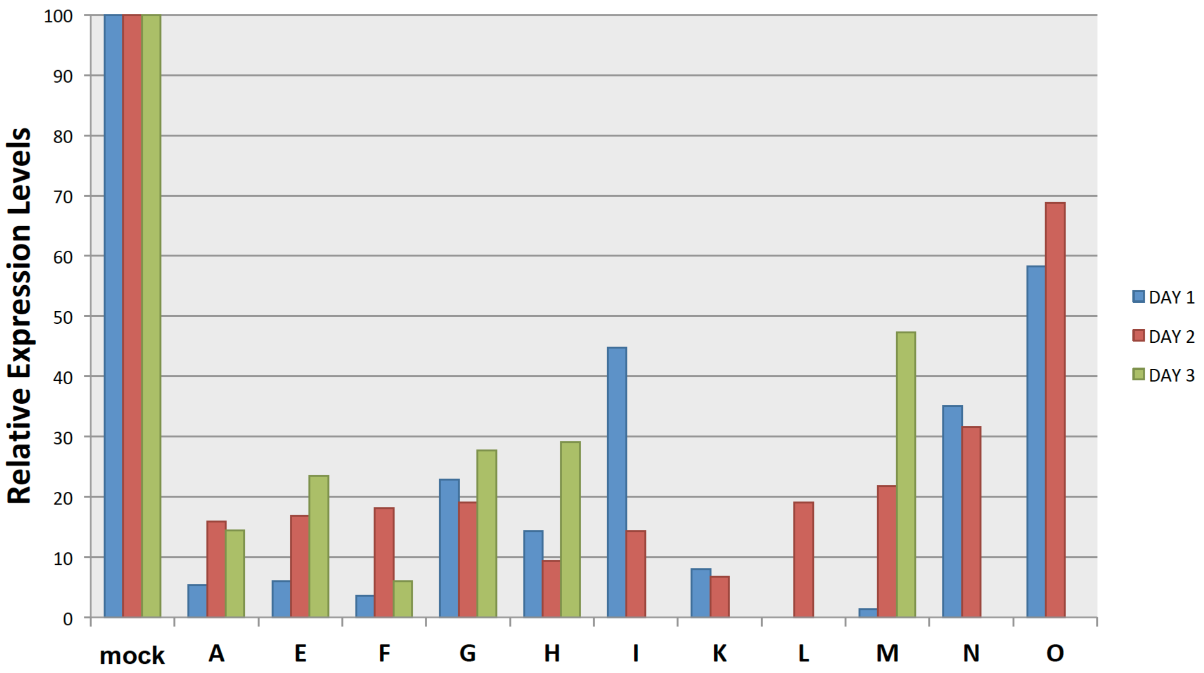

Additional File 1: Fig. S4. Relative embryonic expression of CLANK transcripts over time following pRNAi. cDNA was generated from aged (3-7 h, 28°C) embryos, collected from pRNAi injected females for up to three days post eclosure. mRNA expression levels of the knockdown transcript were monitored via qPCR. Relative expression compared to mock injected embryos (as a percentage out of 100) was calculated and plotted after reactions were normalized via *Nv-rp49* expression. Expression values are an average of biological and technical replicates.

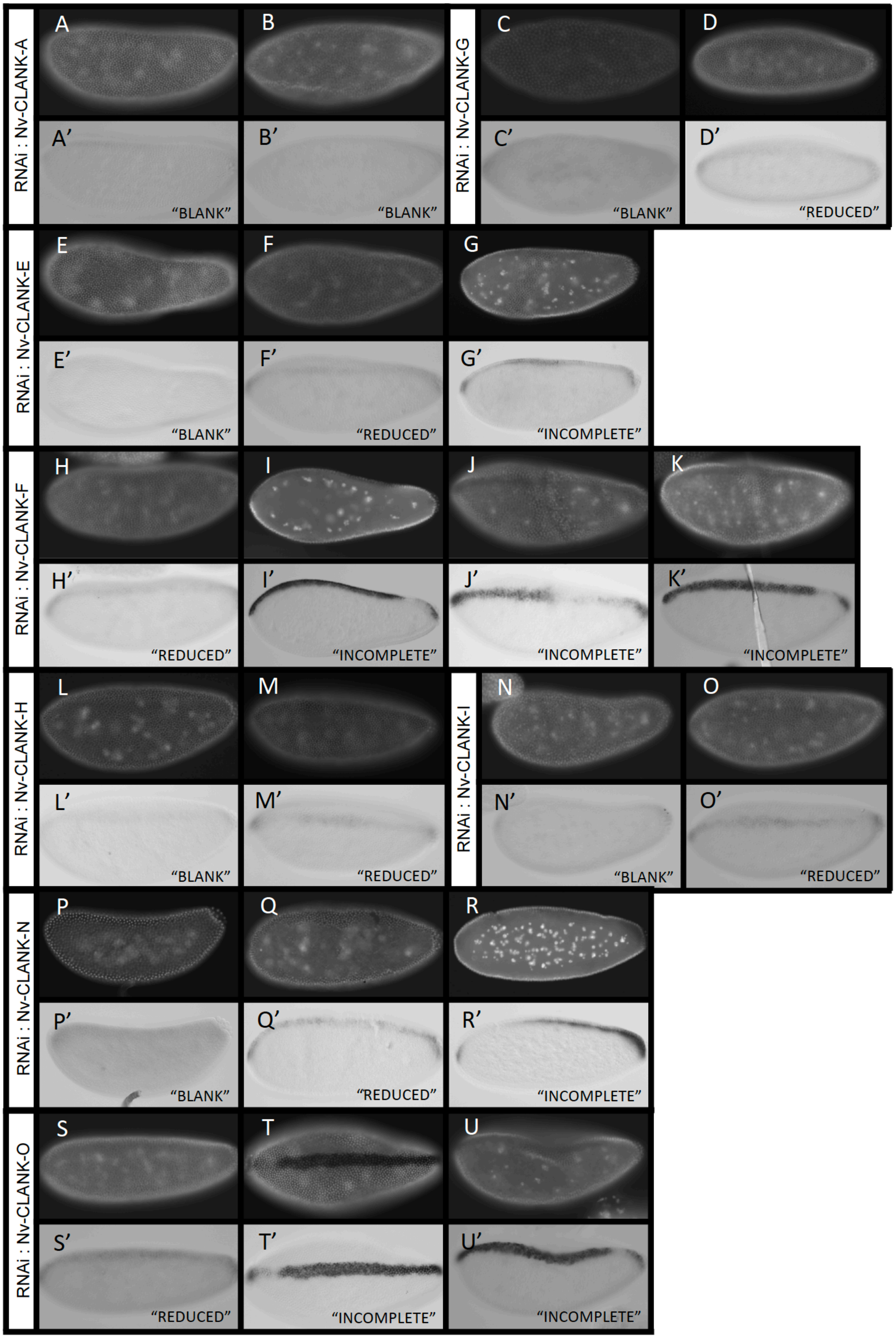

RNAi : Nv-CLANK-A
A
B
A'
"BLANK"
B'
"BLANK"
RNAi : Nv-CLANK-G
C
D
C'
"BLANK"
D'
"REDUCED"
RNAi : Nv-CLANK-E
E
F
G
E'
"BLANK"
F'
"REDUCED"
G'
"INCOMPLETE"
RNAi : Nv-CLANK-F
H
I
J
K
H'
"REDUCED"
I'
"INCOMPLETE"
J'
"INCOMPLETE"
K'
"INCOMPLETE"
RNAi : Nv-CLANK-H
L
M
L'
"BLANK"
M'
"REDUCED"
RNAi : Nv-CLANK-I
N
O
N'
"BLANK"
O'
"REDUCED"
RNAi : Nv-CLANK-N
P
Q
R
P'
"BLANK"
Q'
"REDUCED"
R'
"INCOMPLETE"
RNAi : Nv-CLANK-O
S
T
U
S'
"REDUCED"
T'
"INCOMPLETE"
U'
"INCOMPLETE"

Additional File 1: Fig. S5. Effects of reducing *CLANKs* on *Nv-zen* expression. **A-U'** Altered expression of *Nv-zen* following pRNAi of a *CLANK* of interest in mid-late blastoderm embryos. **A-U** Knockdown embryos stained with DAPI to approximate embryo age. **A'-U'** *In situ* hybridization of knockdown embryos probing for *Nv-zen* expression. Embryos correspond to same embryos in **A-U**. All embryos are oriented with anterior to the left, posterior to the right, dorsal up, and ventral down. **A-B'** *Nv-CLANK-A* pRNAi embryos. **C-D'** *Nv-CLANK-G* pRNAi embryos. **E-G'** *Nv-CLANK-E* pRNAi embryos. **H-K'** *Nv-CLANK-F* pRNAi embryos. **L-M'** *Nv-CLANK-H* pRNAi embryos. **N-O'** *Nv-CLANK-I* pRNAi embryos. **P-R'** *Nv-CLANK-N* pRNAi embryos. **S-U'** *Nv-CLANK-O* pRNAi embryos. Descriptive term of phenotype observed in bottom right corner of *in situ* images.

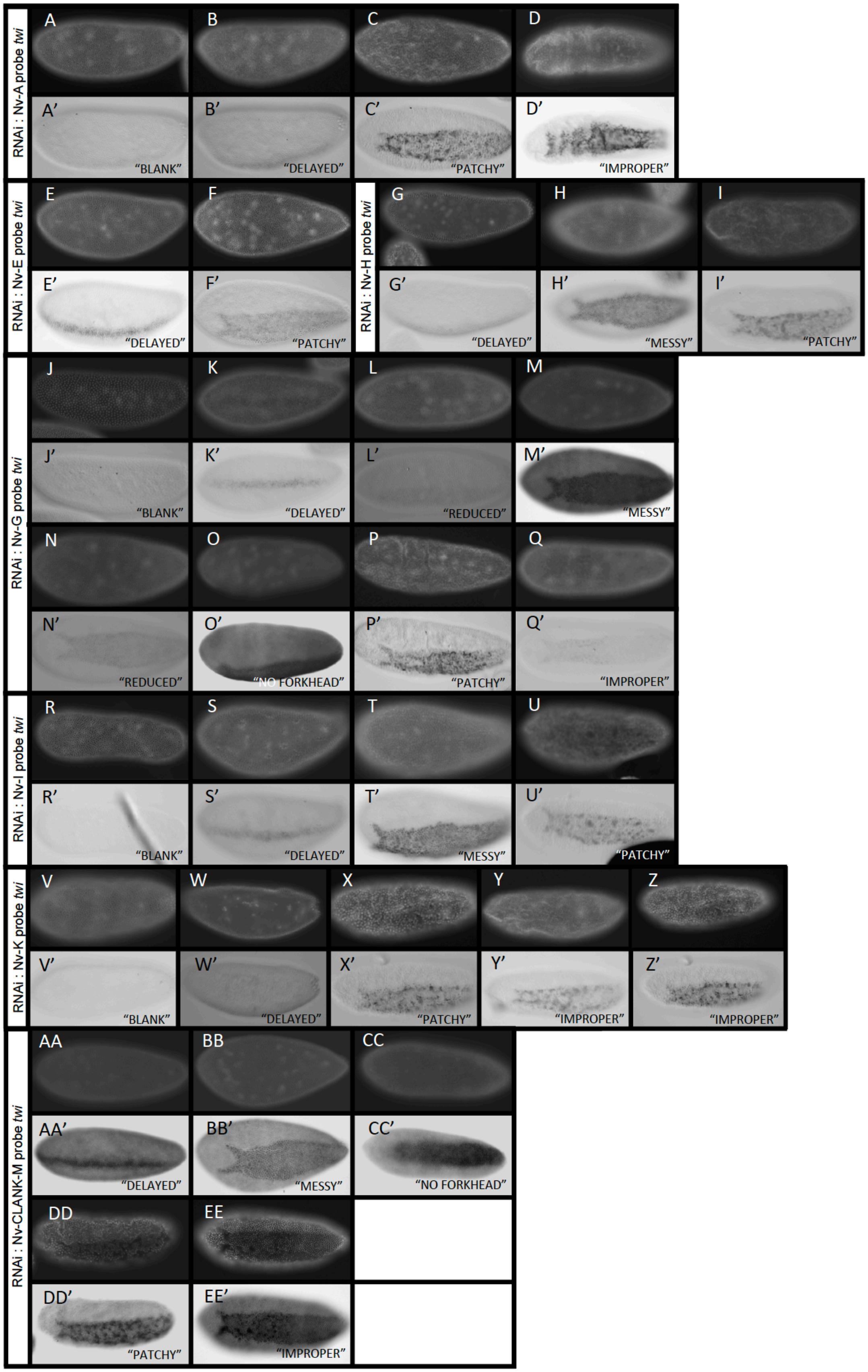
RNAi : Nv-A probe twi
A
B
C
D
A'
"BLANK"
B'
"DELAYED"
C'
"PATCHY"
D'
"IMPROPER"
RNAi : Nv-E probe twi
E
F
E'
"DELAYED"
F'
"PATCHY"
RNAi : Nv-H probe twi
G
H
I
G'
"DELAYED"
H'
"MESSY"
I'
"PATCHY"
RNAi : Nv-G probe twi
J
K
L
M
J'
"BLANK"
K'
"DELAYED"
L'
"REDUCED"
M'
"MESSY"
N
O
P
Q
N'
"REDUCED"
O'
"NO FORKHEAD"
P'
"PATCHY"
Q'
"IMPROPER"
RNAi : Nv-I probe twi
R
S
T
U
R'
"BLANK"
S'
"DELAYED"
T'
"MESSY"
U'
"PATCHY"
RNAi : Nv-K probe twi
V
W
X
Y
Z
V'
"BLANK"
W'
"DELAYED"
X'
"PATCHY"
Y'
"IMPROPER"
Z'
"IMPROPER"
RNAi : Nv-CLANK-M probe twi
AA
BB
CC
AA'
"DELAYED"
BB'
"MESSY"
CC'
"NO FORKHEAD"
DD
EE
DD'
"PATCHY"
EE'
"IMPROPER"

Additional File 1: Fig. S6. Effects of reducing *CLANKs* on *Nv-twi* expression. **A-EE'** Altered expression of *Nv-twi* following pRNAi of a *CLANK* of interest in mid blastoderm to gastrulating embryos. **A-EE** Knockdown embryos stained with DAPI to approximate embryo age. **A'-EE'** *In situ* hybridization of knockdown embryos probing for *Nv-twi* expression. Embryos correspond to same embryos in **A-EE**. All embryos are oriented with anterior to the left, posterior to the right, dorsal up, and ventral down (unless otherwise noted). **A-D'** *Nv-CLANK-A* pRNAi embryos (C/C', D/D' ventral views). **E-F'** *Nv-CLANK-E* pRNAi embryos. **G-I'** *Nv-CLANK-H* pRNAi embryos (H/H' ventral views). **J-Q'** *Nv-CLANK-G* pRNAi embryos (K-N' ventral views). **R-U'** *Nv-CLANK-I* pRNAi embryos (S/S', U/U' ventral views). **V-Z'** *Nv-CLANK-K* pRNAi embryos. **AA-EE'** *Nv-CLANK-M* pRNAi embryos (AA-EE' ventral views). Descriptive term of phenotype observed in bottom right corner of *in situ* images.

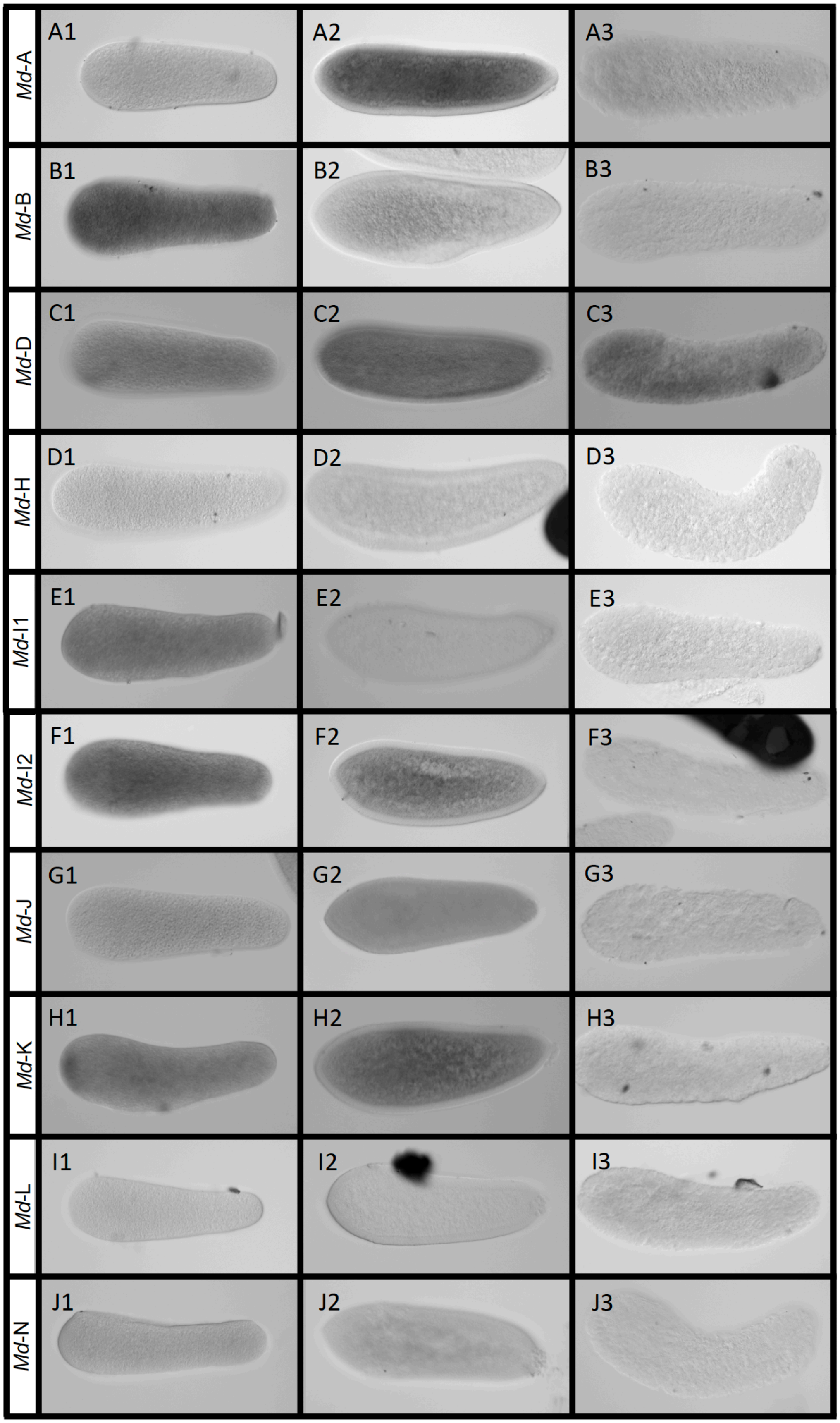
Md-A
A1
A2
A3
Md-B
B1
B2
B3
Md-D
C1
C2
C3
Md-H
D1
D2
D3
Md-I1
E1
E2
E3
Md-I2
F1
F2
F3
Md-J
G1
G2
G3
Md-K
H1
H2
H3
Md-L
I1
I2
I3
Md-N
J1
J2
J3

Additional File 1: Fig. S7. *Melittobia* CLANK candidates lacking differential expression patterns. A1-J3 Expression of *Md-CLANK-A*, *-B*, *-D*, *-H*, *-I1, -I2*, *-J*, *-K*, *-L*, and *-N* from pre-blastoderm through gastrulation. All embryos are oriented with anterior to the left, posterior to the right, dorsal up, and ventral down (except B3, bird's eye dorsal view).
